# Excellent resistive memory characteristics and switching mechanism using a Ti nanolayer at the Cu/TaO_x_ interface

**DOI:** 10.1186/1556-276X-7-345

**Published:** 2012-06-26

**Authors:** Sheikh Ziaur Rahaman, Siddheswar Maikap, Ta-Chang Tien, Heng-Yuan Lee, Wei-Su Chen, Frederick T Chen, Ming-Jer Kao, Ming-Jinn Tsai

**Affiliations:** 1 Department of Electronic Engineering, Chang Gung University, 259 Wen-Hwa 1st Rd., Kwei-Shan, Tao-Yuan, 333, Taiwan; 2 Material and Chemical Research Laboratories, Industrial Technology Research Institute, Hsinchu, 310, Taiwan; 3 Electronic and Opto-Electronic Research Laboratories, Industrial Technology Research Institute, Hsinchu, 310, Taiwan

**Keywords:** Ti nanolayer, Nanoscale, Resistive memory, Nanofilament, Charge-trapping.

## Abstract

Excellent resistive switching memory characteristics were demonstrated for an Al/Cu/Ti/TaO_x_/W structure with a Ti nanolayer at the Cu/TaO_x_ interface under low voltage operation of ± 1.5 V and a range of current compliances (CCs) from 0.1 to 500 μA. Oxygen accumulation at the Ti nanolayer and formation of a defective high-κ TaO_x_ film were confirmed by high-resolution transmission electron microscopy, energy dispersive X-ray spectroscopy, and X-ray photo-electron spectroscopy. The resistive switching memory characteristics of the Al/Cu/Ti/TaO_x_/W structure, such as HRS/LRS (approximately 10^4^), stable switching cycle stability (>10^6^) and multi-level operation, were improved compared with those of Al/Cu/TaO_x_/W devices. These results were attributed to the control of Cu migration/dissolution by the insertion of a Ti nanolayer at the Cu/TaO_x_ interface. In contrast, CuO_x_ formation at the Cu/TaO_x_ interface was observed in an Al/Cu/TaO_x_/W structure, which hindered dissolution of the Cu filament and resulted in a small resistance ratio of approximately 10 at a CC of 500 μA. A high charge-trapping density of 6.9 × 10^16^ /cm^2^ was observed in the Al/Cu/Ti/TaO_x_/W structure from capacitance-voltage hysteresis characteristics, indicating the migration of Cu ions through defect sites. The switching mechanism was successfully explained for structures with and without the Ti nanolayer. By using a new approach, the nanoscale diameter of Cu filament decreased from 10.4 to 0.17 nm as the CC decreased from 500 to 0.1 μA, resulting in a large memory size of 7.6 T to 28 Pbit/sq in. Extrapolated 10-year data retention of the Ti nanolayer device was also obtained. The findings of this study will not only improve resistive switching memory performance but also aid future design of nanoscale nonvolatile memory.

## Background

Recently, many resistive switching random access memory (ReRAM) devices containing oxides such as SrTiO_3_[1-3], Al_2_O_3_[4], NiO_x_[5-7], Na_0.5_Bi _0.5_TiO_3_[8], ZnO [9,10], Ta_2_O_5_[11], ZrO_2_[12-15], GdO_x_[16,17], HfO_x_[18,19], and TiO_x_[21-23] have been reported for future nanoscale nonvolatile memory applications. However, the resistive switching mechanism of ReRAM devices is currently debated. On the other hand, conductive-bridge ReRAM devices with different solid-electrolytes, such as GeSe_x_[24-27], GeS [28,29], Ta_2_O_5_[30,31], ZrO_2_[32], SiO_2_[33], Ag_2_S [34,35], HfO_2_[36,37], SrTiO_3_[38], and Cu-Te/Al_2_O_3_[39] have also been reported by several groups. In these cases, silver (Ag) or copper (Cu) metal can be used as one of the electrodes to mobilize Ag^+^ or Cu^2+^ ions. Under an external bias on Ag or Cu electrode, metallic filament can be formed (or dissolved) into solid-electrolyte films. In general, Cu is a more preferable material than Ag because it is used as an interconnection metal in computer motherboards. High-κ Ta_2_O_5_ is considered the most promising as a resistive switching material. [30,31] Therefore, a resistive switching memory device with a Cu/TaO_x_/W structure could be desirable but also may have the drawback of copper oxidation (CuO_x_) at the Cu/TaO_x_ interface, which can hinder resistive switching characteristics. Moreover, controlling Cu ion transportation and recovery under external bias is difficult. Tada et al. [40] reported resistive switching memory based on a dual layered TiO_x_/TaSiO_x_ solid-electrolyte under a high current compliance (CC) of 800 μA and a large operation voltage of 2.5 V. To prevent CuO_x_ formation as well as promote easier Cu filament formation/dissolution through the high-κ TaO_x_ solid-electrolyte, a Ti nanolayer at the Cu/TaO_x_ interface is a promising approach to design an Al/Cu/Ti/TaO_x_/W resistive switching memory device, which has not been reported to date. Furthermore, Ti has good adhesion behavior and provides a good Cu diffusion barrier. The Gibbs free energies of TiO_2_, Ta_2_O_5_, CuO, and Cu_2_O films at 300 K are −889.5, –760.75, –129.7, and −149.0 kJ/mole, respectively [41], suggesting that the Ti nanolayer can be easily oxidized compared with the other possible materials. Therefore, a Ti nanolayer will consume more oxygen from the TaO_x_ layer and will form TiO_x_/TaO_x_. Consequently, the Cu electrode will not form a CuO layer at the Cu/TaO_x_ interface because of this greater oxygen consumption at the Ti nanolayer. This also has the benefit of easily controlling Cu migration and collection through the resulting higher defective high-κ TaO_x_ solid-electrolyte under external bias. In this study, excellent resistive switching memory characteristics were observed in the proposed Al/Cu/Ti/TaO_x_/W structure with a Ti nanolayer at the Cu/TaO_x_ interface annealed at 350°C in ambient N_2_ compared with a similar structure without a Ti nanolayer. This configuration will be useful for complementary metal-oxide-semiconductor (CMOS) processing after back end of line. The fabricated Al/Cu/Ti/TaO_x_/W structure memory device with a small area of 150 × 150 nm^2^ was observed by high-resolution transmission electron microscopy (HRTEM), X-ray photo-electron spectroscopy (XPS), and energy dispersive X-ray (EDX). In addition, the migration and collection (or formation/dissolution) of Cu ions through the defective TaO_x_ solid-electrolyte under external bias, as well as its switching mechanism, were determined from capacitance-voltage (C-V) hysteresis. Improved resistive switching memory performance of the Al/Cu/Ti/TaO_x_/W structure compared with that of the Al/Cu/TaO_x_/W structure was also obtained, such as repeatable switching cycles with maintenance of a high resistance ratio (approximately 10^4^), long extrapolated program and erase endurance of > 10^6^ cycles, multi-level capability, and extrapolated 10 y data retention. Furthermore, Cu nanofilament diameters were calculated under current compliances (CCs) of 0.1 to 500 μA using a new approach. A large memory size of 28 Pbit/sq in. was achieved with a small nanofilament diameter of 0.17 nm under a CC of 0.1 μA.

## Methods

**Figure 1:**
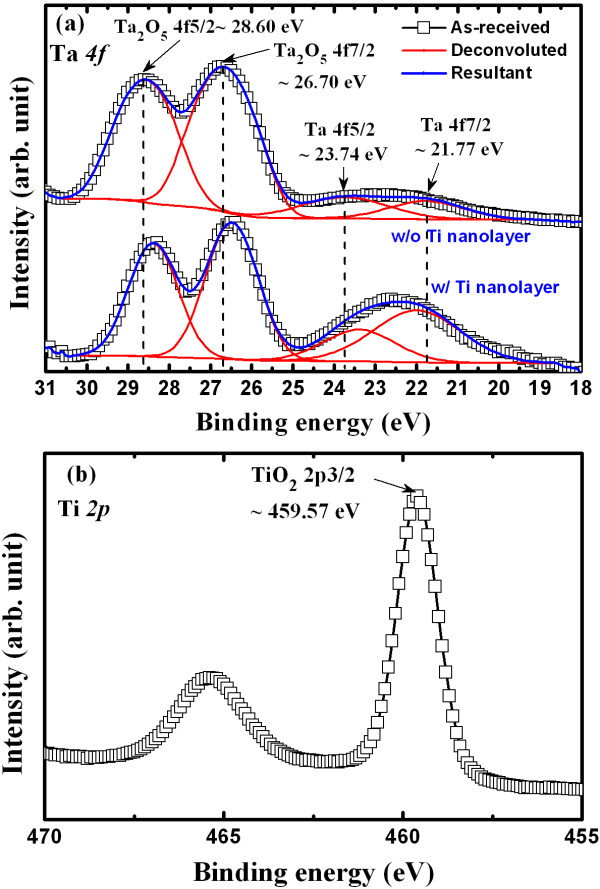
**XPS spectra of (a) Ta*4f*and (b) Ti*2p*core-level electrons.**

Tungsten (W) metal was used as the bottom electrode (BE) and was deposited by sputtering onto SiO_2_/Si substrate. The thickness of the SiO_2_ layer was approximately 200 nm whereas that of the W layer was approximately 100 nm. A further SiO_2_ layer, which had a thickness of approximately 150 nm, was then deposited on the W/SiO_2_/Si substrates. A small device with an area of 150 × 150 nm^2^ was fabricated by lithography. Photoresist was coated on the patterned wafers and both the active and top electrode (TE) regions of the memory device were then exposed for a lift-off process. A high-κ Ta_2_O_5_ solid-electrolyte with a thickness of 18 nm was deposited from pure Ta_2_O_5_ granules using an E-gun evaporator. The resulting high-κ Ta_2_O_5_ solid-electrolyte film was mixed with Ta metal (i.e.,TaO_x_ where x < 2.5), as characterized by XPS. Cu, which provided mobile ions, was deposited at a layer thickness of approximately 50 nm by a thermal evaporator. A 160-nm-thick layer of aluminum (Al) was then deposited *in situ* using the same thermal evaporator to protect the Cu surface layer from oxidation by the external environment. The total thickness of the top electrode (Cu + Al) was approximately 200 nm. Finally, the lift-off process was performed to obtain the resistive switching memory device. Cu mobile ions play a major role in the Al/Cu/TaO_x_/W structure resistive switching memory device (device S1). In this S1 device, Cu is oxidized at the Cu/TaO_x_ interface and hinders the resistive switching memory performance. Therefore, Cu oxidation is expected to be avoided by inserting a Ti nanolayer at the Cu/TaO_x_ interface. A thin Ti layer with a thickness of approximately 3 nm was deposited *in situ* using an E-gun evaporator and Ti granules during the described process to obtain a device with an Al/Cu/Ti/TaO_x_/W structure (device S2). The memory devices were annealed by rapid thermal annealing at 350 °C in ambient N_2_ for 1 min. Oxygen accumulated in the Ti nanolayer and formed a TiO_x_nanolayer, as characterized by XPS. More than twenty resistive switching memory devices were fabricated and measured randomly to determine and compare their memory performance. The microstructures and thicknesses of all layers were investigated by HRTEM at 200 keV. The memory devices were observed by TEM using an FEI Helios-400 s system (FEI Co., Hillsboro, OR, USA) with a Ga^+^ ion source at an operating voltage of 5 kV. All layers and materials were characterized by both XPS and EDX. TaO_x_ and TiO_x_ films were analyzed by XPS using an Al (K_α_) monochrome X-ray source at 1,486.6 eV (Figure 1). The analysis area was 650 μm in diameter, and the base vacuum in the analytic chamber was 1 × 10^−9^ Torr. All spectra were calibrated using a reference C1s peak at 284.6 eV. Memory characteristics, such as current–voltage (I-V), C-V, read endurance, and retention were measured using an HP4156C semiconductor parameter analyzer and HP4284A LCR meter (Agilent Technologies Inc., Santa Clara, CA, USA). The frequency applied during the C-V measurements was 1 MHz. The capacitance was measured in parallel capacitance-conductance mode. The bias applied to the TE and the BE was grounded during I-V measurements, and CC was controlled by an HP4156C analyzer.

## Results and discussion

Figure 1a shows the XPS spectra of Ta *4f* core-level electrons. Peaks were fitted using Gaussian functions. The peak binding energies of Ta_2_O_5_ 4f_7/2_ and Ta_2_O_5_ 4f_5/2_ electrons in the sample without (w/o) Ti were centered at 26.7 and 28.6 eV, respectively. For the device with (w/) Ti, the peak binding energies of Ta_2_O_5_ 4f_7/2_ and Ta_2_O_5_ 4f_5/2_ electrons were centered at 26.48 and 28.39 eV, respectively. The w/ Ti sample showed slightly lower binding energies than the w/o Ti sample, attributed to oxygen accumulation in the Ti layer from the Ta_2_O_5_ layer. Chang et al. [42] previously reported a larger binding energy shift of approximately 4.5 eV after heat treatment of the Ti/Ta_2_O_5_ layers due to oxygen accumulation in the Ti nanolayer (i.e., formation of TiO_2_ or TiO_x_ film). The binding energies of the Ta 4f_7/2_ and Ta 4f_5/2_ electrons for the w/o Ti sample were centered at 21.77 and 23.74 eV, respectively, while those of the w/ Ti sample were centered at 21.99 and 23.39 eV, respectively. These results suggest that the high-κ Ta_2_O_5_ film in both structures contained Ta metal (i.e.,TaO_x_ where x < 2.5). The peak height ratios of the Ta 4f_7/2_, Ta 4f_5/2_, and Ta_2_O_5_ 4f_5/2_ core levels for the w/o Ti samples with respect to the Ta_2_O_5_ 4f_7/2_ peak height were 0.03, 0.03, and 0.77, respectively, while those for the w/ Ti samples were 0.27, 0.16, and 0.77, respectively. This also suggests that the Ta content was higher in the w/ Ti samples than in the w/o Ti samples. Furthermore, the TiO_2_ 2p_3/2_ binding energy in the w/ Ti samples was 459.57 eV (Figure 1b), which is close to that reported in previous studies [43,44]. These results indicate that a higher Ta metal content was present in the w/ Ti sample because of oxygen migration from the TaO_x_ film to the Ti film, resulting in TiO_x_/TaO_x_ bilayers. Because of this oxygen accumulation property of the Ti nanolayer, the Cu layer was expected to be protected from oxidation at the Cu/TaO_x_ interface. In addition, the high-κ TaO_x_ solid-electrolyte is expected to be more defective, which will lead to improve resistive switching memory characteristics in the Al/Cu/Ti/TaO_x_/W structure compared with the Al/Cu/TaO_x_/W structure. The resulting Cu oxidation and memory characteristics are explained below.

**Figure 2:**
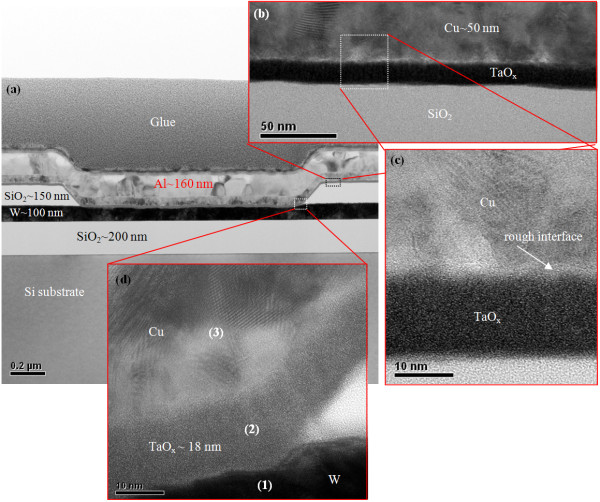
**TEM and HRTEM images of Al/Cu/TaO**_**x**_**/W structure.** (**a**) TEM image of an Al/Cu/TaO_x_/W structure. Enlarged images with (**b**) scale bars of 50 nm and (**c**) 10 nm. (**d**) HRTEM image from (**a**) with a scale bar of 10 nm.

**Figure 3:**
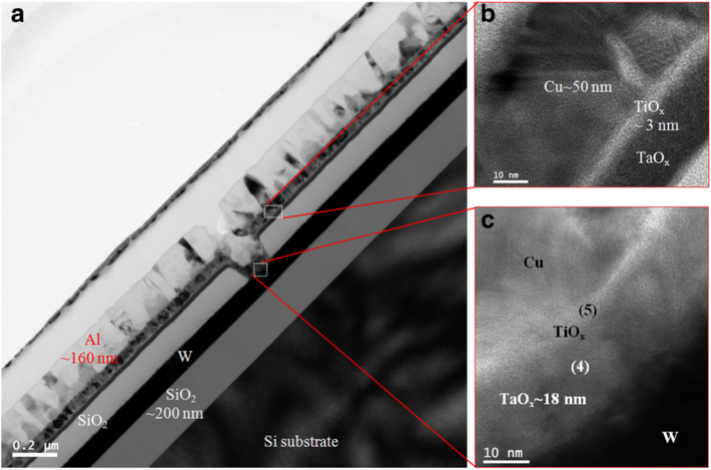
**TEM image of an Al/Cu/Ti/TaO**_**x**_**/W structure.** (**a**). HRTEM images on (**b**) outside and (**c**) inside of the structure shown in (**a**).

**Figure 4:**
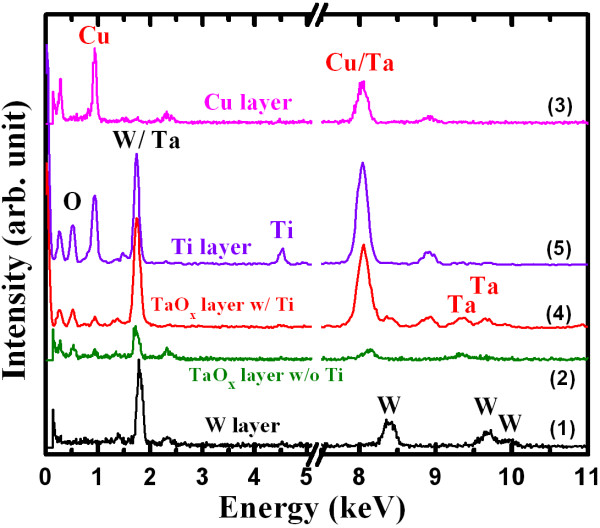
**EDX spectra from Figures**2**and**3. Numbers indicate corresponding positions in Figures 2 and 3.

Figure 2a shows a typical TEM image of an Al/Cu/TaO_x_/W resistive switching memory device (S1) of approximately 1 × 1 μm in size. The thicknesses of the W, Cu, and Al layers were approximately 100, 50, and 160 nm, respectively. A rough interface between the Cu and TaO_x_ layer was observed by HRTEM (Figure 2b,c), attributed to the formation of copperoxide in an approximately 3-nm-thick CuO_x_:TaO_x_ mixture layer at the Cu/TaO_x_ interface. Considering the Gibbs free energies [41], the formation of CuO_x_ at the Cu/TaO_x_ interface is not possible. However, a CuO_x_:TaO_x_ mixture was expected to form at the Cu/TaO_x_ interface during the device fabrication process. We have attempted to overcome this problem but in our experience to date it is difficult to avoid because the Cu first diffuses into the TaO_x_ layer and then intermixes, forming a CuO_x_:TaO_x_ layer. Therefore, Ti barrier layer for Cu diffusion is generally used for interconnection of CMOS. Because of the observed thin (<3 nm) intermixing layer and the existence of the same materials (Cu and TaO_x_) on both sides of this interfacial layer, the exact composition at the Cu/TaO_x_ interface was not easy to determine in this report. Further study is needed to investigate the formation and composition of the Cu/TaO_x_ interface. The high-κ TaO_x_ film had a thickness of approximately 18 nm and appeared amorphous (Figure 2d). Figure 3a shows a TEM image of the device with a Ti nanolayer at the Cu/TaO_x_ interface (S2). The device size was approximately 150 × 150 nm. All layers were covered well at the active and outer regions of the device (Figure 3b,c). Oxygen accumulated in the Ti layer as expected, resulting in an approximately 3-nm-thick TiO_x_nanolayer on the high-κ TaO_x_ solid-electrolyte. This accumulated oxygen originated from the TaO_x_ layer and formed a more defective TaO_x_ solid-electrolyte. The composition of all layers was also confirmed by EDX (Figure 4). The spectra in Figure 4 are labeled with corresponding positions in Figures 2 and 3. The observed Cu, W/Ta, W, Ta, and Ti signals had energies of approximately 0.94, 1.72, 8.38, 9.36, and 4.54 keV, respectively. The Cu count in the TaO_x_ layer w/ Ti nanolayer was lower than that in the layer w/o Ti nanolayer at an energy of 0.94 keV (108 vs. 228, respectively). The oxygen counts at an approximate energy of 0.52 keV were 17, 238, 129, and 378 for W, TaO_x_ w/o Ti, TaO_x_ w/ Ti, and pure Ti layers, respectively. The oxygen count in the Ti nanolayer was the highest of any layer in the resistive switching memory device. These findings suggest that the Ti nanolayer is easily oxidized, which is in agreement with the Gibbs free energy, resulting in the formation of TiO_x_ on the TaO_x_ solid-electrolyte. TiO_x_nanolayer formation at the Cu/TaO_x_ interface improved resistive switching memory characteristics as described below.

**Figure 5:**
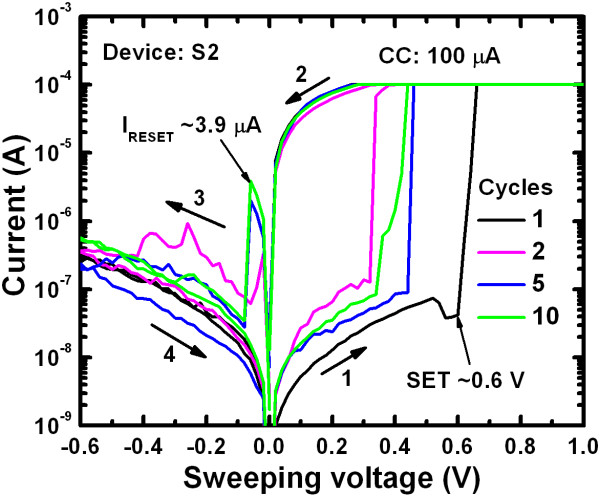
**Typical I-V hysteresis loop under a CC of 100 μA for the Al/Cu/Ti/TaO**_**x**_**/W structure.** The RESET current increases with the number of cycles.

Figure 5 shows a typical I-V hysteresis loop for the Al/Cu/Ti/TaO_x_/W resistive switching memory device (S2). Ten consecutive switching cycles at a CC of 100 μA are indicated by arrows 1 to 4. The pristine device had a high resistance state (HRS) with a leakage current of 5.26 nA at a read voltage (V_read_) of +0.1 V. When the bias voltage applied to the top electrode was larger than the SET voltage of + 0.6 V, Cu filament formed in the TaO_x_ solid-electrolyte and induced a low resistance state (LRS) through a chemical reduction process (Cu^z+^ + ze^–^ → Cu, where *z* is 1 or 2). By applying a negative voltage of approximately −0.6 V, the Cu filament was dissolved or reverted to the TE through a chemical oxidation process (Cu → Cu^z+^ + ze^–^), and the device was reverted to a HRS. The SET voltage varied between 0.2 and 0.6 from cycle-to-cycle and device-to-device (data not shown). It has previously been shown that a higher formation voltage (e.g. >5 V) is not needed to observe resistive switching characteristics of the pristine device [45]. It is interesting to note that the value of the RESET current (I_RESET_) increased as the number of switching cycles increased. No RESET current was observed for the first switching cycle of the pristine device. These results suggested that the filament diameter is very thin (or unstable) during the first switching cycle. When the number of switching cycles was increased to 10, the RESET current reached approximately −3.9 μA, and a stable filament was formed, suggesting that Cu migration was controlled by the TiO_x_ layer. In this case, the initial filament formation of a pristine device depends on a few switching cycles rather than the formation voltage, which is commonly needed for a ReRAM device. Therefore, this provides an advantage for future real applications of Cu metallic filament resistive switching memory devices. The present device also only requires very low operation voltage of ±1 V with a current of only a few nanoamperes. Although it has been reported that Cu nanofilament has been successfully formed in solid-electrolyte, Cu migration in solid-electrolyte has not been described in the literature to date. To address this point, C-V hysteresis characteristics were measured to obtain an understanding of the solid-electrolyte under external bias.

**Figure 6:**
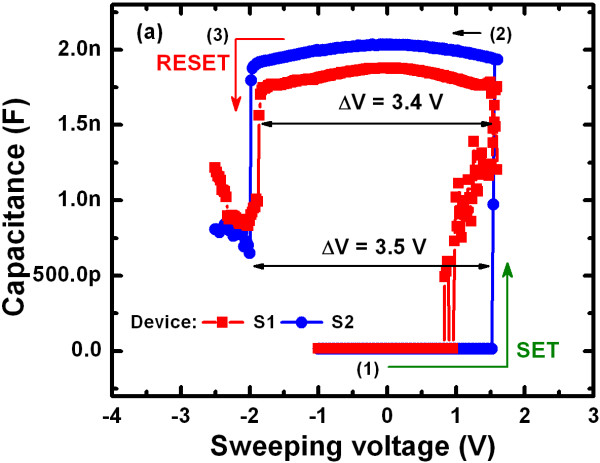
C-V hysteresis characteristics for w/ and w/o Ti nanolayer capacitors.

Figure 6 shows the typical C-V hysteresis characteristics for both resistive switching memory devices under sweeping voltages from −1 V → +1.6 V → –2.5 V. The typical size of the device (A) was 8 × 8 μm. The switching cycle is indicated by arrows 1 → 3. The capacitances of pristine S1 and S2 devices at HRS were approximately 14.5 and 13.5 pF, respectively, at a V_read_ of + 0.1 V. The capacitances at LRS were very high at approximately 1.87 and 2.03 nF for the S1 and S2 devices, respectively, under SET operation at a V_read_ of + 0.1 V. This result was likely due to the movement of Cu ions through defect sites, which were trapped in the defective high-κ TaO_x_ solid-electrolyte (i.e., Cu filament formation). The charge-trapping density was calculated from C-V hysteresis characteristics using the Equation 1 below [46].

(1) $$N_{\text{charge}}=\frac{\Delta V.C_{\text{LRS}}}{q.A}\text{,}$$

where ‘q’ (= 1.602 × 10^−19^ C) is the electronic charge and ‘A’ is the area of the resistive switching memory device. The hysteresis memory windows (ΔV) were approximately 3.4 and 3.5 V for the S1 and S2 devices, respectively. The charge-trapping densities of the S1 and S2 resistive switching memory devices were approximately 6.2 × 10^16^ and 6.9 × 10^16^/cm^2^, respectively. C-V was measured using a 4284A LCR meter (Agilent Technologies Inc., Santa Clara, CA, USA). This system does not have capacitance compliance function. To confirm the capacitance values, more than fifty devices on each wafer were measured randomly. The Ti nanolayer of the Cu/TaO_x_ device (S2) had a higher charge-trapping density due to trapping of more Cu ions at the defect sites of the TaO_x_ solid-electrolyte, which improved the resistive switching performance as described below.

**Figure 7:**
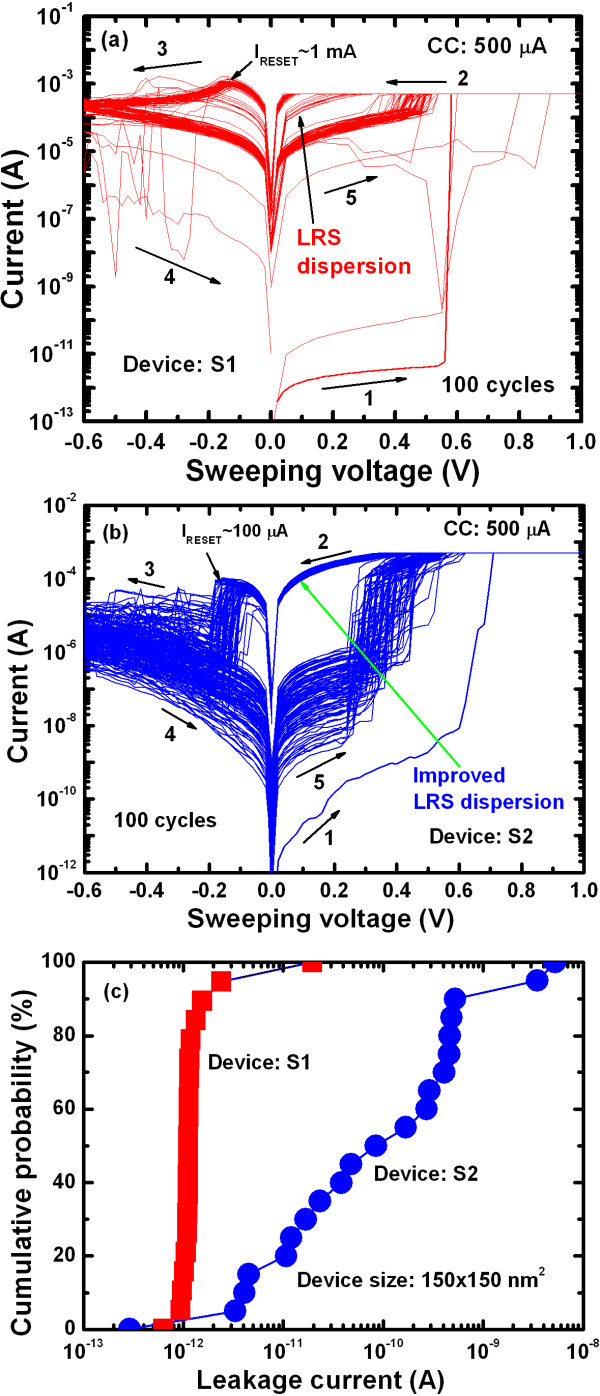
**Typical I-V hysteresis loop of S1 and S2 devices and cumulative probability of leakage currents.** Typical I-V hysteresis loop with 100 consecutive cycles under a CC of 500 μA for (**a**) S1 and (**b**) S2 devices. (**c**) Cumulative probability of leakage currents for S1 and S2 devices.

**Figure 8:**
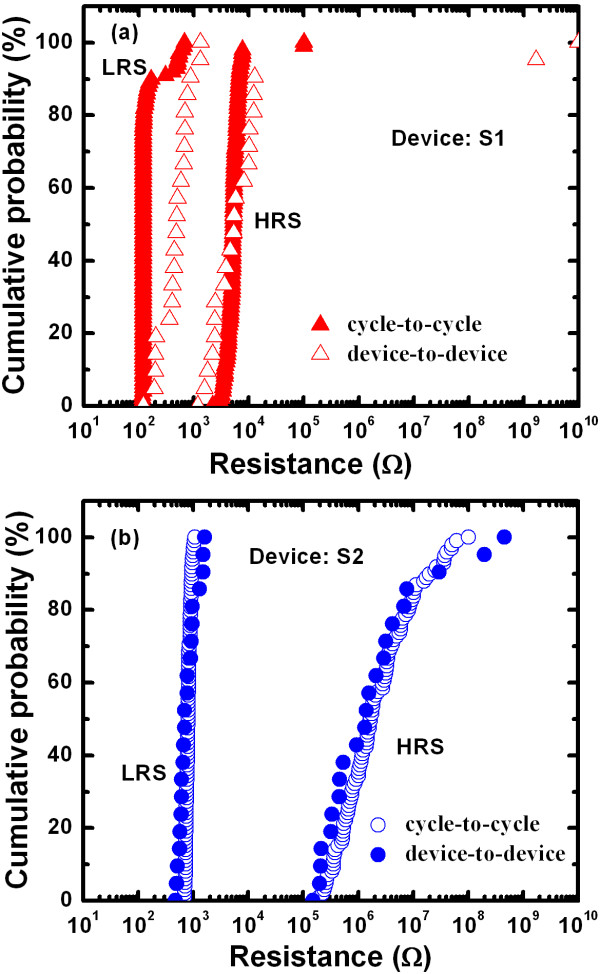
**The cumulative probability plots of LRS and HRS.** Cumulative probability plots of LRS and HRS under a CC of 500 μA for (**a**) Al/Cu/TaO_x_/W and (**b**) Al/Cu/Ti/TaO_x_/W devices with cycle-to-cycle and device-to-device measurements.

**Figure 9:**
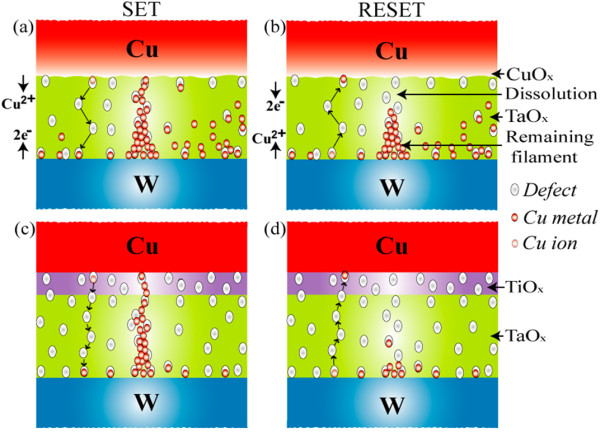
**The schematic view of filament formation/dissolution.** Schematic view of filament formation/dissolution under SET and RESET operations for w/ and w/o Ti nanolayer resistive switching memories. Filament formation (**a**) and dissolution (**b**) for w/o Ti nanolayer devices. Controllable filament formation (**c**) and dissolution (**d**) for w/ Ti nanolayer resistive switching memory devices.

Figure 7 shows the typical I-V hysteresis loops for both resistive switching memory devices. One hundred consecutive switching cycles are indicated by arrows 1 to 5 under a CC of 500 μA. The S1 memory devices showed a leakage current of approximately 1.5 pA at a V_read_ of + 0.1 V for a pristine device, lower than that of the S2 devices (23 pA) (Figure 7a,b). Average values (± standard deviations (SD)) of the leakage currents for the S1 and S2 memories were 1.5 (4.7) and 180 (10.7) pA, respectively (Figure 7c). Because of a higher charge-trapping density (Figure 6), the average leakage current of the S2 memory devices was higher than that of the S1 devices. The HRS currents at a V_read_ of +0.1 V increased after the second cycle to approximately 32 and 0.57 μA for S1 and S2 devices, respectively. The lower HRS current of the S2 devices compared with that of the S1 devices was attributed to a longer filament length remaining in the S1 devices. This suggests that the recovery of Cu ions in the S2 devices was caused by a higher amount of defects in the TaO_x_ layer. The SET voltages of the S1 devices showed larger variation (+0.3 to 0.9 V) compared with those of the S2 devices (0.23 to 0.5 V). In addition, the I_RESET_ of the S2 devices was lower than that of the S1 devices (100 vs. 1,000 μA, respectively); this was because of the control of Cu migration by the Ti nanolayer at the Cu/TaO_x_ interface. The average device-to-device LRS values (± SD) of the S1 and S2 devices were 5.5 × 10^2^ (3.2 × 10^2^) and 8.4 × 10^2^ (3.4 × 10^2^) Ω, respectively, and the average HRS (± SD) values were 5.9 × 10^3^ (4.0 × 10^3^) and 3.2 × 10^7^ (10.4 × 10^7^) Ω, respectively (Figure 8a,b). For cycle-to-cycle, the average LRS values (± SD) of the S1 and S2 devices were 1.6 × 10^2^ (1.3 × 10^2^) and 8.2 × 10^2^ (0.88 × 10^2^) Ω, respectively, whereas the average HRS (± SD) values were 7.1 × 10^3^ (1.34 × 10^4^) and 7.4 × 10^6^ (1.5 × 10^7^) Ω, respectively. The resulting resistance ratios (HRS/LRS) of the S1 and S2 devices were approximately 10 and 3.8 × 10^4^ for device-to-device and 44 and 9 × 10^3^ for cycle-to-cycle, respectively. The resistance ratio of the S1 device was smaller than that of the S2 (10 vs. 10^4^, respectively), which was attributed to the smaller gap (i.e., dissolved filament length) between the Cu electrode and remaining filament. Because of Cu ion migration under external bias, the filament formation/dissolution can be explained with the following hypothesis. Considering the charge-trapping phenomenon shown in Figure 6, the Cu ions (as positive charges) are transported through the defect sites into the high-κ TaO_x_ solid-electrolyte under external bias. These ions are then trapped in the defect sites (starting from the W BE) and consequently neutralized by electrons from the BE. This causes metallic Cu filament to grow from the BE and form a conical-type metallic filament between the BE and TE (Figure 9a), a different mechanism to that recently reported for ZnO [10]. Under RESET operation, the Cu filament then starts to dissolve from the Cu/TaO_x_ interface (Figure 9b) because the electric field at the Cu/TaO_x_ interface will be higher as a result of its conical-shape. In this case, under negative bias to the TE, the filament Cu ions are transported through the similar defect sites into the high-κ TaO_x_ solid-electrolyte. The Cu ions then either capture electrons from the TE, resulting in neutralization or return to the Cu electrode. In this case, the Cu ions will partially return to the Cu electrode because of the copper oxide present at the Cu/TaO_x_ interface. On the other hand, the Cu filament forms, and ions migrate through the TaO_x_ solid-electrolyte easily because of the higher charge-trapping density (i.e., defect sites), as shown in Figure 9c. It is clear that the observed lower diameter of the Cu filament is due to the Ti nanolayer at the Cu/TaO_x_ interface controlling the migration of Cu ions under SET operation. Therefore, one of the major reasons that the control of the Cu ion mobilization is easier in the device with Ti is because of the higher defective TaO_x_ solid-electrolyte. Under RESET operation, either the filament is almost dissolved (Figure 9d) or the gap length (i.e., the length between the Cu electrode and the remaining filament, or length of filament dissolution) in the S2 devices is longer than that of the S1 devices. The longer gap length of the S2 devices produced a higher resistance ratio (approximately 10^4^ vs. 10, respectively) than that exhibited by the S1 devices because of the easier dissolution of the Cu filament through the defective TaO_x_ solid-electrolyte. Through observation of several switching cycles (Figure 8), we found that the standard deviation of HRS for the S2 devices was larger than that for the S1 devices (1.5 × 10^7^ vs. 1.34 × 10^4^ Ω, respectively), which was attributed to the longer gap length. However, the SD of LRS for the S2 devices was smaller than that for the S1 devices (88 vs. 130 Ω, respectively) because of the Ti nanolayer controlling Cu ion migration. Therefore, LRS dispersion was better in S2 devices compared with S1 devices. It is also interesting to note that under a CC of 500 μA, the LRS of the S2 devices was higher than that of the S1 devices (840 vs. 550 Ω, respectively) because of the series resistance effect of Ti acting as a Cu barrier layer. Notably, the observed longer gap length in the S2 devices was attributed to either easier dissolution of the Cu filament or higher amount of electron injection from the Cu electrode through the Ti nanolayer. It has been reported that the electron affinities of Ta_2_O_5_ and TiO_2_ films with respect to the Si conduction band are 3.77 [47] and 3.15 eV, respectively [32,47]. Therefore, our results indicate that the barrier height at the Cu/Ta_2_O_5_ interface was reduced because of the Ti nanolayer, increasing electron injection from the Cu electrode. Similar barrier height lowering at the TiO_x_/ZrO_2_ interface and improved resistive switching memory performance were reported by Li et al. [48]. These findings suggest that Cu recovery from the filament through the TaO_x_ solid-electrolyte is easier in the device with Ti. The defective high-κ TaO_x_ solid-electrolyte, Cu ion migration control, and lower barrier height of the Ti nanolayer at the Cu/TiO_x_ interface in the Al/Cu/Ti/TaO_x_/W structure were therefore expected to maintain repeatable and improved resistive switching memory characteristics, as described below.

**Figure 10:**
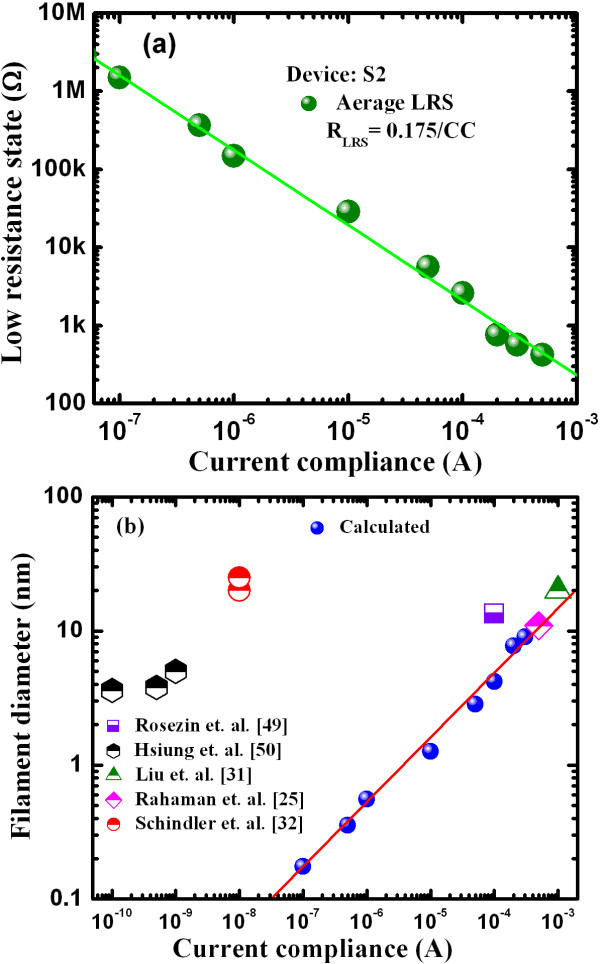
**The average LRS and filament diameter.** (**a**)Average LRS under current compliances from 0.1 to 500 μA and (**b**) filament diameter versus current compliances for the S2 structure.

Figure 10 shows the dependence of average LRS with CC ranging from 0.1 to 500 μA for the S2 devices. The LRS decreased linearly with increasing CC. The observed control of LRS by the device operation current was independent of the device size, which confirms the filamentary conduction mechanism proposed for the present resistive switching memory device. The average (median) value of LRS under a CC of 1 μA was approximately 147 (145) kΩ for a typical device of 150 × 150 nm. All LRS data were captured from several devices within the same cycle number. As a comparison, the LRS value of Ag-GeSe solid-electrolyte is approximately 140 kΩ at a CC of 1 μA [28]. The resistance at LRS can be expressed in relation to CC by Equation 2

(2) $$R_{\text{LRS}}=\frac{0.175}{CC}\text{.}$$

The average LRS determined for the present S2 devices was slightly higher than that previously reported (0.14 /CC), which was due to both the series resistance of the Ti nanolayer and use of a different solid-electrolyte. Considering the cylindrical shape of the Cu filament, its diameter can be calculated from Equation 3

(3) $$R_{\mathrm{LRS}}=\frac{\rho_{\text{filament}}.L}{\Phi }\text{,}$$

(4) $$\Phi =\frac{\pi .D^{2}}{4}\text{,}$$

(5) $$D=\sqrt{\frac{4.L.\rho_{\text{filament}}}{\pi .R_{\text{LRS}}}}\text{,}$$

where *L* is the length (thickness of high-κ TaO_x_ solid-electrolyte, approximately 18 nm), *ρ*_filament_ is the resistivity (approximately 200 μΩ.cm [49]), Ф is the cross-sectional area, and D is the diameter of the Cu filament. Using R_LRS_ and CC as obtained from equations 2 and 5, respectively, the filament diameter was found to decrease linearly with CC (Figure 10). The nanoscale filament diameter was 10.4 to 0.17 nm as the CC decreased from 500 to 0.1 μA. These calculated filament diameters were generally consistent with those in some reports [26,32,50] but were slightly different than others in the literature [33,51], which may be due to different solid-electrolytes and structures used. The observed small diameter of 1.7 Å at a small CC of 0.1 μA indicates that this device will be scalable beyond the atomic scale in the future. Under a CC of 500 μA, a memory size of 7.6 Tbit/sq in will be obtained, higher than that previously reported (60 Gbit/sq in. [35]). If a low current operation of 0.1 μA is achieved, then an even larger memory size of 28 Pbit/sq in. will be obtained, a significant benefit to future high-density nonvolatile memory applications.

**Figure 11:**
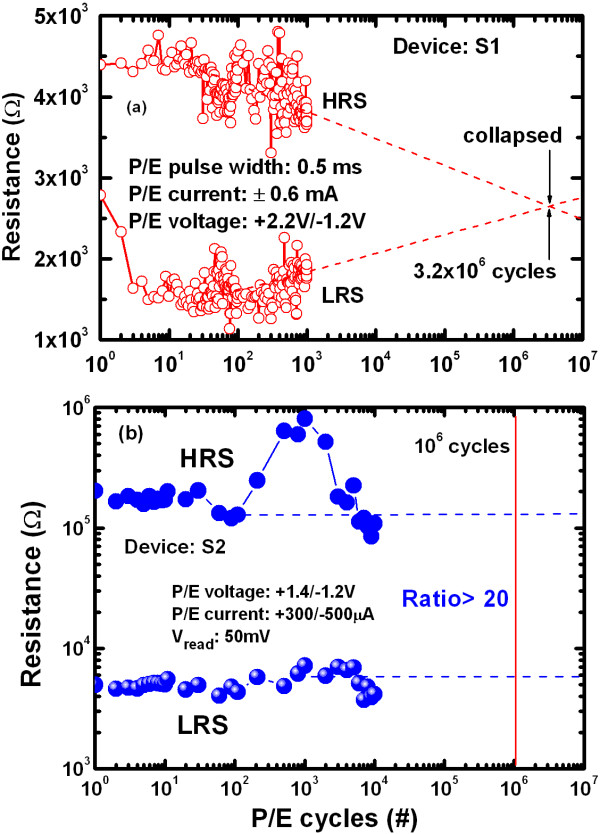
P/E endurance characteristics for (a) S1 and (b) S2 resistive switching memory devices.

**Figure 12:**
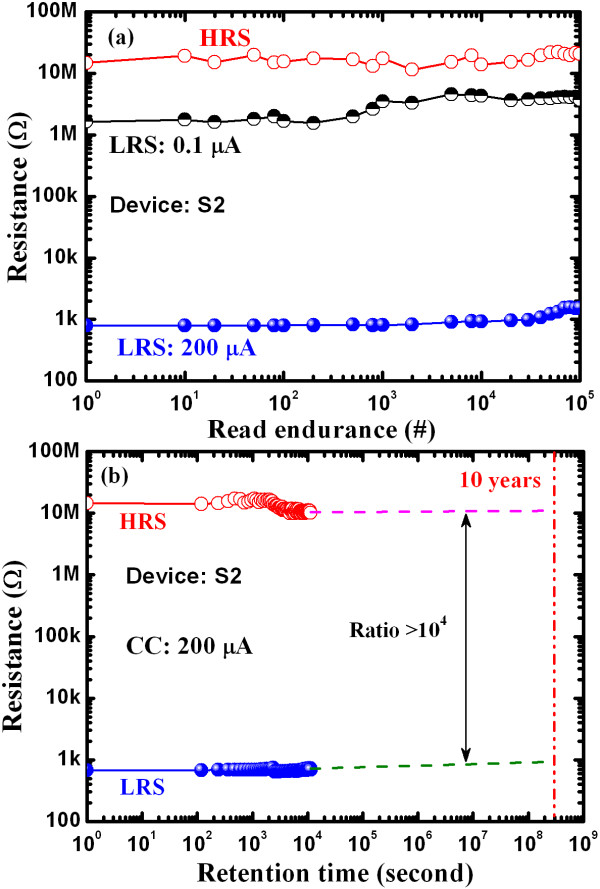
**The typical read endurance and data retention characteristic for the device with Ti nanolayer.** (**a**)Typical read endurance and (**b**) data retention characteristics for the device with Ti nanolayer at the Cu/TaO_x_interface (S2). The device is capable of operation at a low current compliance of 0.1 μA.

Figure 11 shows typical program/erase (P/E) endurance characteristics of the resistive switching memory devices. The S2 device had improved switching cycles, with P/E voltage of +1.4/–1.2 V, pulse width of 500 μs, and P/E current of +300/–500 μA (Figure 11b). An extrapolated P/E endurance of > 10^6^ cycles with a resistance ratio of > 20 was observed for the S2 devices, whereas the S1 device collapsed at 3.2 × 10^6^ cycles. This result was attributed to the Ti nanolayer at the Cu/TaO_x_ interface in the S2 devices. Excellent read endurance characteristics of the Al/Cu/Ti/TaO_x_/W memory device were obtained even after >10^5^ cycles under a read voltage of +0.1 V and CCs of 0.1 and 200 μA (Figure 12a). Under a CC of 200 μA, a high resistance ratio of > 10^4^ was obtained after 10^5^ cycles, whereas under a low CC of 0.1 μA, a smaller ratio of 2 to 3 was obtained and there was no collapse even after 10^5^ cycles. Figure 12b shows typical data retention characteristics as well as 10 y extrapolated data retention under a CC of 200 μA, performed at 25 °C. A high resistance ratio of > 10^4^ was obtained after the extrapolated data retention, which will be of great advantage in future nanoscale low power nonvolatile resistive switching memory applications.

## Conclusions

In summary, superior resistive switching memory performance of an Al/Cu/Ti/TaO_x_/W structure in a CC range of 0.1 to 500 μA was demonstrated compared with that of an Al/Cu/TaO_x_/W structure. The resistive switching mechanism was elucidated and explained by a schematic model, correlated with charge-trapping phenomena observed from C-V hysteresis characteristics. These findings suggest that the formation/dissolution of Cu nanofilament can be controlled by insertion of a Ti nanolayer at the Cu/TaO_x_ interface or using a defective high-κ TaO_x_ solid-electrolyte. The high-κ TiO_x_ and TaO_x_ films were amorphous as observed by HRTEM. The oxygen accumulation properties of the Ti nanolayer at the Cu/TaO_x_ interface were confirmed by EDX and XPS analyses. Good uniformity with large resistance ratio of approximately 10^4^, long P/E endurance of > 10^6^ cycles, MLC operation, and 10 y data retention were observed for the devices with Ti nanolayer at the Cu/TaO_x_ interface. The LRS increased linearly with decreasing CC in the range 500 to 0.1 μA, which resulted in a decrease of the Cu nanofilament diameter from 10.4 to 0.17 nm. These results suggest that a large memory size of 28 Pbit/sq in. at a small CC of 0.1 μA will be possible for the present devices for future nanoscale (0.17 nm) nonvolatile memory applications.

## Competing interests

The authors declare that they have no competing interests.

## Authors’ contributions

SZR carried out the device fabrication, measurement, and data analysis under the instruction of SM. TCT performed the XPS measurements. HYL, WSC, FTC, MJK, and MJT performed via structure design and fabrication. All the authors contributed to the preparation and revision of the manuscript. All authors read and approved the final manuscript.
